# Changes in left ventricular structure and function associated with renal transplantation: a systematic review and meta‐analysis

**DOI:** 10.1002/ehf2.13283

**Published:** 2021-03-15

**Authors:** Luke C. Pickup, Jonathan P. Law, Ashwin Radhakrishnan, Anna M. Price, Charalampos Loutradis, Toby O. Smith, Nicola C. Edwards, Richard P. Steeds, Jonathan N. Townend, Charles J. Ferro

**Affiliations:** ^1^ Birmingham Cardio‐Renal Group, Institute of Cardiovascular Sciences University of Birmingham Birmingham UK; ^2^ Nuffield Department of Orthopaedics, Rheumatology and Musculoskeletal Sciences University of Oxford Oxford UK

**Keywords:** Echocardiography, Magnetic resonance imaging, Heart failure, Meta‐analysis, Kidney transplantation, Cardiomyopathy

## Abstract

**Aims:**

This study aimed to examine if the cardiac changes associated with uraemic cardiomyopathy are reversed by renal transplantation.

**Methods and results:**

MEDLINE, Embase, OpenGrey, and the Cochrane Library databases were searched from 1950 to March 2020. The primary outcome measure was left ventricular mass index. Secondary outcome measures included left ventricular dimensions and measures of diastolic and systolic function. Studies were included if they used any imaging modality both before and after successful renal transplantation. Data were analysed through meta‐analysis approaches. Weight of evidence was assessed through the Grading of Recommendations Assessment, Development and Evaluation system. Twenty‐three studies used echocardiography, and three used cardiac magnetic resonance imaging as their imaging modality. The methodological quality of the evidence was generally poor. Four studies followed up control groups, two using cardiac magnetic resonance imaging and two using echocardiography. Meta‐analysis of these studies indicated that there was no difference in left ventricular mass index between groups following transplantation {standardized mean difference −0.07 [95% confidence interval (CI) −0.41 to 0.26]; *P* = 0.67}. There was also no difference observed in left ventricular ejection fraction [mean difference 0.39% (95% CI −4.09% to 4.87%); *P* = 0.86] or left ventricular end‐diastolic volume [standardized mean difference −0.24 (95% CI −0.94 to 0.45); *P* = 0.49]. Inconsistent reporting of changes in diastolic dysfunction did not allow for any meaningful analysis or interpretation.

**Conclusions:**

The evidence does not support the notion that uraemic cardiomyopathy is reversible by renal transplantation. However, the evidence is limited by methodological weaknesses, which should be considered when interpreting these findings.

## Introduction

Over half of deaths in end‐stage kidney disease (ESKD) are due to cardiovascular disease; the age‐corrected relative risks are extreme, reaching over 100‐fold in younger patients.^1^ The majority of these deaths are not due to myocardial infarction as a result of coronary atheroma but due to heart failure and sudden cardiac death.^2^, ^3^, ^4^ Consistent with this observation, treatments for traditional cardiovascular risk factors such as hypertension and elevated cholesterol are relatively ineffective in this population.^4^, ^5^, ^6^ These observations can be explained by the near‐universal syndrome of uraemic cardiomyopathy in patients with ESKD.^7^, ^8^ Left ventricular hypertrophy is the cardinal feature of uraemic cardiomyopathy, in addition to ventricular dilatation and both systolic and diastolic dysfunction. Histologically, myocytes are severely hypertrophied with myocardial disarray and diffuse interstitial fibrosis.^9^ As renal function declines, these features become more prevalent and are present in up to 90% of those requiring renal replacement.^10^ Such changes are strongly linked to cardiovascular outcomes with the presence of left ventricular hypertrophy associated with increased mortality in both transplant recipients and those requiring haemodialysis.^11^, ^12^ The gold standard for the treatment of ESKD is renal transplantation.^10^ The associated improvement in glomerular filtration rate reduces cardiovascular risk below that of those on waiting lists.^13^ However, cardiovascular risk still remains higher than healthy individuals of the same age and sex with transplant recipients displaying a three‐fold increased risk.^14^

The restoration of renal function associated with renal transplantation improves many factors thought to cause uraemic cardiomyopathy. As a result, it is generally assumed that kidney transplantation reduces left ventricular mass index (LVMI) and volumes and improves diastolic and systolic function.^15^, ^16^ This assertion is based on the reduction of LVMI reported in small echocardiographic studies.^15^, ^16^ However, echocardiography is not a reliable or reproducible method for the measurement of LVMI, especially when there are large changes in loading such as before and after haemodialysis.^17^ As a result, cardiac magnetic resonance imaging (CMR) is now accepted as the gold‐standard imaging modality for patients with ESKD.^7^ Despite this, review articles continue to state that uraemic cardiomyopathy is reversed by renal transplantation. These articles will not cite any references, cite small, uncontrolled studies using either echocardiography or radionucleotide ventriculography‐gated blood pool (multigated acquisition scan) scans, or refer to other review articles.^8^, ^15^, ^18^, ^19^, ^20^

The aim of this study was to perform the first systematic review and meta‐analysis to establish if the features of uraemic cardiomyopathy are reversible following successful renal transplantation.

## Methods

A Preferred Reporting Items for Systematic Reviews and Meta‐Analyses‐compliant systematic review was conducted and was registered with the International Prospective Register of Systematic Reviews (PROSPERO; http://www.crd.york.ac.uk/prospero/, Reference CRD42018115359).^21^ Published and unpublished articles and conference proceedings registered on or before 20 March 2020 were searched. The electronic databases used to search the published literature were MEDLINE, Embase, OpenGrey, and the Cochrane Library (clinical trials database and database of systematic reviews). All searches were limited to adult human studies. Reference lists of all pertinent review papers and eligible studies were reviewed. The search terms used are presented for the MEDLINE search in *Table* *1*. These were modified for the specific databases searched.

**Table 1:** Search strategy for MEDLINE database

1. exp Adult/
2. chronic kidney disease.mp. or exp Renal Insufficiency, Chronic/
3. exp Kidney Failure, Chronic/
4. 1 or 2 or 3
5. exp Echocardiography/or echocardiogram*.mp
6. Heart Ventricles/dg [Diagnostic Imaging]
7. exp Myocardial Perfusion Imaging
8. Magnetic resonance imaging.mp. or exp Magnetic Resonance Imaging/
9. 5 or 6 or 7 or 8
10. exp Kidney Transplantation
11. exp Renal Replacement Therapy/
12. exp Renal dialysis/
13. 10 or 11 or 12
14. exp Hypertrophy, Left Ventricular/
15. cardiomyopathy.mp. or exp Cardiomyopathies/
16. uremic cardiomyopathy.mp.
17. exp Ventricular Remodeling/
18. exp Ventricular Dysfunction, Left/
19. exp Heart Failure, Diastolic/
20. left ventricular mass.mp.
21. 14 or 15 or 16 or 17 or 18 or 19 or 20
22. 4 and 9 and 13 and 21
23. remove duplicates from 22

### Inclusion criteria

All full‐text English‐language articles assessing changes in LVMI, before and after successful renal transplant, using any form of imaging technique were included. Single‐subject case reports, comments, letters, editorials, guidelines, or review papers were excluded. Studies were also excluded if participants received more than one organ type.

### Study selection

Two reviewers (L.C.P. and J.P.L.) independently reviewed all titles and abstracts generated from the search strategy. Following this initial screening process, the full texts of eligible articles were reviewed independently by each author against the predefined eligibility criteria.

### Critical appraisal

All papers were critically appraised independently by two reviewers (L.C.P. and A.R.). This appraisal was conducted using the Newcastle–Ottawa Scale.^22^ A maximum score of 9 points can be awarded based on participant selection, comparability, and study outcome including follow‐up.

The Grading of Recommendations Assessment, Development and Evaluation (GRADE) system was adopted to evaluate the quality of the evidence across studies for pooled analyses.^23^

### Outcome measures and data extraction

Two reviewers (L.C.P. and J.P.L.) extracted data into a pre‐constructed table. Information gathered included number of participants, age range, sex distribution, dialysis modality, immunosuppression regime, and time to follow‐up after transplantation. The primary outcome measure was LVMI. Secondary outcome measures were left ventricular dimensions, measures of diastolic and systolic function. Any disagreement regarding study eligibility, data extraction, methodological quality, and GRADE assessment between reviewers was resolved through discussion until consensus was reached.

### Statistical analysis

Statistical analysis was conducted using Review Manager 5.0 for Apple (Nordic Cochrane Centre, Copenhagen, Cochrane Collaboration, 2008). Statistical heterogeneity was assessed by *χ* ^2^ and *I* ^2^. If *χ* ^2^ was greater than *P* = 0.10 and the *I* ^2^ statistic indicated that heterogeneity was present (>20%), a random‐effects statistical model was adopted to calculate mean difference or standardized mean difference (SMD) between groups. When *χ* ^2^and *I* ^2^ values demonstrated low heterogeneity, a fixed‐effects model was adopted.^24^ Where meta‐analysis was not possible because of insufficient data, a narrative approach was adopted.

## Results

### Search strategy results

**Figure 1:**
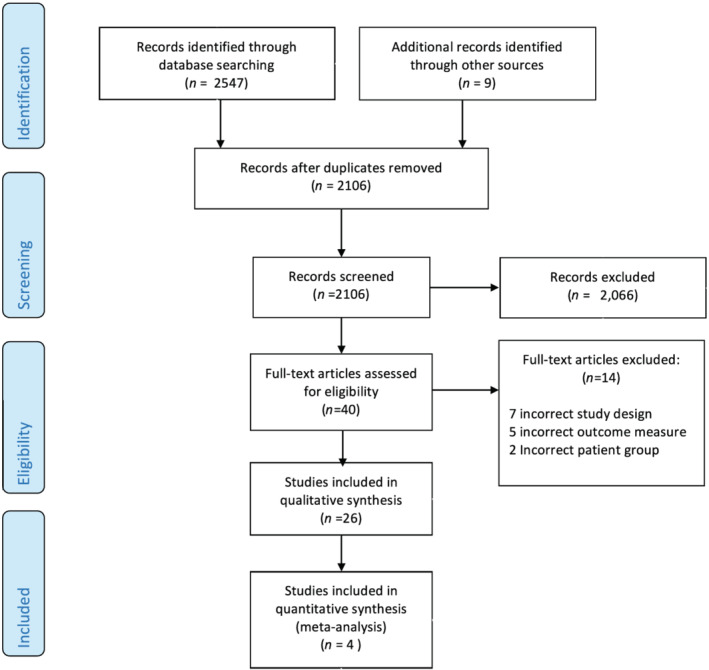
Preferred Reporting Items for Systematic Reviews and Meta‐Analyses flow diagram.

The results of the search strategy are summarized in *Figure* *1*. A total of 2547 potentially relevant citations were identified, with 26 being eligible for inclusion. The characteristics and outcomes of the 26 included studies are presented in *Table* *1*.

### Methodological appraisal

There were 23 studies that used echocardiography.^25^, ^26^, ^27^, ^28^, ^29^, ^30^, ^31^, ^32^, ^33^, ^34^, ^35^, ^36^, ^37^, ^38^, ^39^, ^40^, ^41^, ^42^, ^43^, ^44^, ^45^, ^46^, ^47^ Of these studies, eight were retrospective echocardiographic data collected as part of routine clinical practice.^25^, ^31^, ^34^, ^37^, ^38^, ^45^, ^46^, ^47^ Their methodological quality was largely poor (*Table* *1*). One study was classified as fair,^35^ and none were classified as good. Two studies recruited control groups, which were followed up, both consisting of individuals receiving haemodialysis.^27^, ^35^ Assessor blinding was only employed in two studies,^28^, ^30^ and none used a sample size calculation.

Three studies employed CMR, two were classified as good^48^, ^49^ and one as fair.^50^ In each, recipients were recruited from local transplant waiting lists. No study performed a sample size calculation designed to detect change in LVMI. Prasad *et al*.,^48^ however, used a sample size calculation powered to detect changes in adiponectin levels. Assessor blinding was employed in all CMR studies.^48^, ^49^, ^50^ In one study, the indication for initial CMR was routine clinical practice,^49^ and in the remaining two, CMR was conducted for research purposes.^48^, ^50^

The length of follow‐up across all the studies varied from 1 week to 5 years, with the most common follow‐up time point being 12 months.

### Study population

In total, 1998 renal transplant recipients were included of which 1229 were male. The pooled weighted mean age was 50 years (range 16–85 years). Fourteen studies reported type of transplant with a total of 840 live donor transplants and 377 deceased donor transplants.^25^, ^27^, ^29^, ^31^, ^34^, ^35^, ^36^, ^38^, ^40^, ^42^, ^43^, ^44^, ^48^, ^50^ A total of 1531 recipients were reported to be receiving renal replacement therapy in 24 studies.^25^, ^26^, ^27^, ^29^, ^30^, ^31^, ^32^, ^33^, ^35^, ^36^, ^37^, ^38^, ^39^, ^40^, ^41^, ^43^, ^44^, ^45^, ^46^, ^47^, ^48^, ^49^, ^50^ In total, 127 control patients were followed up in four studies.^27^, ^35^, ^48^, ^49^ Two CMR^48^, ^49^ studies recruited both recipients and controls from local transplant waiting lists. Comparisons between the groups at baseline showed that there was no difference in age, sex, systolic blood pressure, or history of ischaemic heart disease. Two echocardiographic studies also recruited controls. De Lima *et al*. ^27^ recruited 74 unselected ESKD patients on regular haemodialysis, 17 who were subsequently transplanted. There was no significant difference between those transplanted and those who remained on dialysis in terms of age, gender, race, or duration of haemodialysis. No data regarding blood pressure or prior cardiac disease were presented. Keven *et al*.^35^ also recruited both transplant recipients and randomly selected controls receiving haemodialysis. There were no significant differences reported between recipients and controls in terms of age, sex, and systolic blood pressure. There was also no recorded ischaemic heart disease in either group. Further two studies recruited controls who were studied at a single time point; in both cases, however, these were healthy controls.^36^, ^50^ Findings of all studies are summarized in *Table* *2*.

**Table 2:** Selected data for all studies

Author	Subjects	Age (years)	Follow‐up	RRT—modality^a^ Duration (months)	Primary outcome measure findings	NOS	Comments
Hayer *et al*.^50^ UK CMR	Transplant group: 24 live donor recipients Control group: 18 healthy controls	Transplant group: 46 ± 13 Control group: 49 ± 17	2 months	HD 11 PD 3 Duration 13 (IQR 8–33)	No significant reduction in LVMI (g/m^2^) from baseline 89 ± 38 to follow‐up 83 ± 23	Fair	Prospective Blinded Controlled
Hamidi *et al*.^30^ Iran 2D Echo^b^	25 recipients on HD	44.64 ± 13.91	1 month	HD 25 Duration 56. ± 9.7	Significant reductions in LVMI (g/m^2^) −73.82 ± 11.6, *P* < 0.001, and relative wall thickness 0.056 ± 0.023, *P* = 0.021	Poor	Prospective Single blinded Non‐controlled
Prasad *et al*.^48^ UK CMR	Transplant group: 39 live donor recipients Control group: 43 on local waiting list	Transplant group: 46.5 ± 12.4 Control group: 55.5 ± 11	12 months	Transplant group: HD 27 PD 12 Control group: HD 31 PD 12 Duration NS	No difference in LVMI (g/m^2^) change at 1 year between recipients −1.98 ± 5.5 and waiting list patients −0.36 ± 5.7 g/m^2^; *P* = 0.44	Good	Prospective Single blinded Controlled
Hewing *et al*.^31^ Germany 2D Echo^b^	31 recipients	44 year range: 19–85	Median 19 months	HD 23 Duration 33.5 (IQR 10.0–72.3)	Significant reduction in LVMI (g/m^2^) 111. 2 (IQR 88.7–150.6) to 103.8 (IQR 78.4–113.8); *P* = 0.001. No change observed in LV diastolic function	Poor	Retrospective Non‐blinded Non‐controlled
An *et al*.^25^ Korea 2D Echo^b^	767 recipients	45.0 ± 11.5	1 week 1 year 5 years	HD 495 PD 108 Duration NS	Significant reductions in LVMI (g/m^2^) at 1 and 5 years compared with pre‐transplant and 1 week; *P* < 0.001. Baseline 129.1 (IQR 103.0–161.6), 1 week 130.4 (IQR 103.7–161.6), 1 year 119.9 (IQR 96.5–150.4), and 5 years 110.0 (IQR 90.4–137.2); *P* < 0.001	Poor	Retrospective Non‐blinded Non‐controlled
Hawwa *et al*.^47^ USA 2D Echo^b^	232 recipients	54 ± 12	422 days (median)	HD 163 PD 31 Duration 26 (IQR 8–24)	Significant reduction in LVMI (g/m^2^) pre‐transplant 132 ± 46 and post‐transplant 125 ± 42; *P* = 0.32	Poor	Retrospective Non‐blinded Non‐controlled
Deng *et al*.^28^ USA 2D Echo^b^	48 recipients with no history of MI, cardiomyopathy, CHF, arrhythmias, or OSA	Range (36–67)	6 months	NS NS	Significant reduction in LVMI (g/m^2^) from 104.00 ± 16.47 to 95.50 ± 21.44; *P* = 0.043	Poor	Prospective Single blinded Non‐controlled
Salerno *et al*.^45^ Italy 2D Echo^b^	104 recipients, two alternative immunosuppression strategies; CNI + EVE (28) or CNI + MMF (76)	CNI + EVE: 47.5 ± 13.1 CNI + MMF: 47.8 ± 12.1	36 months	CNI + EVE: RRT 28 Duration 48 ± 37.2 CNI + MMF: RRT 76 Duration 39.6 ± 37.2	No significant difference between immunosuppression groups. Both showed significant reductions in LVMI (g/m^2^) at 3 years in everolimus group 126.5 ± 46.4 to 121.9 ± 39.4 and in the mycophenolate group 116.6 ± 38.3 to 113 ± 28.9; *P* < 0.05	Poor	Retrospective Non‐blinded Non‐controlled
Vaidya *et al*.^46^ USA 2D Echo^b^	105 recipients with ≥1 year of CKD prior to Tx	53.8 ± 12.3	Mean 2.2 years	RRT 87 Duration 36 ± 36	57 participants had significant LVMI (g/m^2^) decrease, mean difference −37.2 ± 31.3, and 48 had no regression mean difference 15.7 ± 17.1. The extent of the LVMI before transplant was the only predictor of LVMI regression odds ratio 1.50 (95% CI 1.26–1.80)	Poor	Retrospective Non‐blinded Non‐controlled
Souza *et al*.^42^ Brazil 2D Echo^b^	40 live donor recipients	31.6 ± 12.7	1 month 3 months 6 months	NS Duration NS	Significant reduction in LVMI (g/m^2^) from baseline 131.48 ± 38.93, to 1 month 126.41 ± 29.45, *P* < 0.05, to 3 months 128.81 ± 30.71 and 6 months 113.03 ± 29.99 (*P* = 0.02 comparison between 6 months and baseline. No significant difference between other follow‐up times and baseline)	Poor	Prospective Non‐blinded Non‐controlled
Namazi *et al*.^39^ Iran Echo modality NS	47 recipients with no history of cardiovascular disease	Range 23–56	4 months	HD 16 PD 4 NS	Significant reduction in LVMI (g/m^2^) from baseline 120 to 110 (SD not given); *P* = 0.002	Poor	Prospective Non‐blinded Non‐controlled
Patel *et al*.^49^ UK CMR	Transplant group: 25 transplant recipients Control group: 25 patients transplant waiting list	Transplant group: 45.9 ± 14.4 Control group: 52.7 ± 10.4	Mean 1.8 (±0.9)	Transplant group: HD 10 Duration 36 ± 36 Control group: HD 12 Duration 28 ± 31	No difference in LVMI change (%/year) between recipients and those who remained on the waiting list, 2.75 ± 9.1 vs. 3.6 ± 16.7; *P* = 0.10	Good	Prospective Single blinded Controlled
Keven *et al*.^35^ Turkey 2D Echo^b^	Transplant group: 28 recipients on HD Control group: 23 controls on HD	34 ± 9	12 months	Transplant group: HD 23 Duration 40 ± 35 Control group: HD 23 Duration 52 ± 20	No change in LVMI (g/m^2^) between transplant 132 ± 38 and HD 145 ± 38; *P* < 0.05	Fair	Prospective Non‐blinded Controlled
Iqbal *et al*.^34^ Bangladesh 2D Echo^b^						Poor	Retrospective Non‐blinded Non‐controlled
‐ Group 1	22 recipients	31 ± 9	3 months	NS Duration 5 ± 1.2	LVMI (g/m^2^) reduced at 3 months from 379 ± 114 to 248 ± 58 g/m^2^ (*P* < 0.001)		
‐ Group 2	30 recipients	31 ± 8	3 months 6 months 12 months	NS Duration 7 ± 3	LVMI (g/m^2^) reduced significantly from baseline 275 ± 91, at 3 months 191 ± 38, 6 months 173 ± 39, and 12 months 159 ± 26; *P* < 0.001		
Hernández *et al*.^43^ Spain 2D Echo^b^	60 divided based on the presence of LVH at baseline	LVH: 52 ± 12 No LVH: 48 ± 12	19 months	HD 43 PD 17 Duration 12 (IQR 6–24)	52% (23) of participants with no LVH at baseline developed LVH or >20% increase in LVMI at follow‐up; 22% (8) participants with LVH at baseline showed regression to normal at follow‐up	Poor	Prospective Non‐blinded Non‐controlled
Montanaro *et al*.^38^ USA Echo modality NS	23 recipients without diabetes	43 ± 10	24 months	HD 17 PD 7 Duration 33 ± 12	LVMI (g/m^2^) reduced at 24 months from 161.4 ± 48.2 to 122.1 ± 27.7 (*P* < 0.007)	Poor	Retrospective Non‐blinded Non‐controlled
Ferreira *et al*.^29^ Brazil 2D Echo^b^	24 recipients on RRT	33.5 ± 10.0	3 months 6 months 12 months	HD 21 PD 3 Duration 23 (range 9–119)	LVMI (g/m^2^) reduced at 12 months from 164.6 ± 47.0 to 130.5 ± 39.8 (*P* = 0.004). The incidence of LVH decreased from 75% to 52.1% 12 months after transplant	Poor	Prospective Non‐blinded Non‐controlled
Sahagun‐Sanchez *et al*.^41^ Mexico 2D Echo^b^	13 recipients on HD	33.64 ± 10.13	3 months 4 months	HD 13 Duration 35.5 (SD NS)	Reduction in LVMI (g/m^2^) from baseline 102.8 ± 27.7 to 3 months 83.5 ± 18.1 and 4 months 71.5 ± 16.2; *P* = 0.001	Poor	Prospective Non‐blinded Non‐controlled
McGregor *et al*.^37^ UK 2D Echo^b^	67 recipients on RRT	38.3 (18.7–64.5)	4 months	RRT 67 Duration NS	No significant change in LVMI (g/m^2^) from baseline 143 (range 61–48) to 4 months 145 (range 62–37) (*P* = 0.71)	Poor	Retrospective Non‐blinded Non‐controlled
Hernandez *et al*.^44^ Spain 2D Echo^b^	38 on RRT, stratified according genotype DD or ID + II of intron 16 of the ACE gene	DD: 46.2 ± 4.1 ID + II: 45.2 ± 2.9	6 months 12 months	RRT 38 Duration DD: 32.3 ± 10.7 ID + II: 26.4 ± 7.3	LVMI increased at 12 months in those with DD genotype from 166.6 ± 10.4 to 201.5 ± 21.6; *P* < 0.05. There was no change in LVMI in the ID + II groups 181.3 ± 9.1 to 176.9 ± 9.4; *P* > 0.05	Poor	Prospective Non‐blinded Non‐controlled
Palfrey *et al*.^40^ Canada 2D Echo^b^	102 recipients	37 ± 12	12 months	HD 72 PD 27 Duration 15 ± 15	LVMI (g/m^2^) reduced from baseline 158 ± 39 to 1 year 132 ± 39; *P* < 0.001	Poor	Prospective Non‐blinded Non‐controlled
De Lima *et al*.^27^ Brazil 2D Echo^b^	Transplant group: 17 live donor recipients Control group: 36 on HD	Transplant group: 44 ± 13 Control group: 40.5 ± 10	15 months	HD 74 Duration minimum 12 months	No change in LVMI (g/m^2^) in recipients 156.7 ± 51.3 vs. 132.9 ± 31.0, *P* > 0.05, or controls 170.6 ± 50.8 vs. 155.6 ± 43.1, *P* > 0.05	Poor	Prospective Non‐blinded Controlled
De Castro *et al*.^26^ Italy 2D Echo^b^	23 non‐diabetic recipients on HD	39.1 ± 13.7	1 year	HD 23 Duration 15 ± 14.3	LVMI (g/m^2^) decreased from 157.78 ± 53.5 to 108.1 ± 19.5 (*P*‐value not stated)	Poor	Prospective Non‐blinded Non‐controlled
Huting^32^ Germany 2D Echo^b^	24 recipients on HD	47 ± 12	Mean 41 ± 30 months	HD 24 Duration 50 ± 29	No change in LVMI (g/m^2^) from baseline 175 ± 48 to follow‐up 171 ± 49; *P* = 0.05	Poor	Prospective Non‐blinded Non‐controlled
Larsson *et al*.^36^ Sweden M‐mode^c^	Transplant group: 27 recipients with juvenile onset diabetes. Control group: 27 healthy men	Transplant group: 33 range (27–45) Control group: 26 ± 2	Transplant group: 6 months 13 months 44 months Control group: single echo	HD 6 Duration NS	LVMI (g/m^2^) decreased from baseline 176 ± 51, to 6 months 143 ± 44, 13 months 133 ± 44, and 44 months 111 ± 22; *P* < 0.01	Poor	Prospective Non‐blinded Controlled
Ikaheimo *et al*.^33^ Finland M‐mode^c^	13 recipients on HD	31 (20–50)	9 months	13 Duration NS	LVMI (g/m^2^) decreased from baseline pre‐HD session 197.7 ± 44.8 and post‐HD session 143.5 ± 47.3 to 143.5 ± 47.3, *P* = 0.001, after transplant	Poor	Prospective Non‐blinded Non‐controlled

ACE, angiotensin‐converting enzyme; CHF, congestive heart failure; CI, confidence interval; CKD, chronic kidney disease; CMR, cardiac magnetic resonance imaging; CNI, calcineurin inhibitor; D, deletion; EVE, everolimus; HD, haemodialysis; I, insertion; IQR, inter‐quartile range; LV, left ventricular; LVH, left ventricular hypertrophy; LVMI, left ventricular mass index; NOS, Newcastle–Ottawa score; NS, not stated; OSA, obstructive sleep apnoea; PD, peritoneal dialysis; RRT, renal replacement therapy; SD, standard deviation; Tx, transplantation.

^a^ 2D Echo: acquisition of two‐dimensional images of cardiac structures.

^b^ M‐mode: acquisition of monodimensional view of cardiac structures along a single ultrasound line.

^c^ RRT indicates number receiving dialysis where a specific modality is not specified.

Aetiology of ESKD was reported in 12 studies,^25^, ^27^, ^29^, ^30^, ^31^, ^33^, ^35^, ^39^, ^41^, ^47^, ^48^, ^50^ with glomerulonephritis being the most commonly reported aetiology. Five studies excluded patients with ischaemic heart disease or congestive cardiac failure,^25^, ^28^, ^30^, ^39^, ^50^ and a further two^32^, ^41^ only included patients who were asymptomatic from cardiovascular disease. Prasad *et al*.^48^ reported that 10% of transplanted patients had undergone coronary revascularization. Vaidya *et al*.^46^ reported that 43% of their cohort had a diagnosis of coronary artery disease, and McGregor *et al*.^37^ indicated that 84% of participants had a dilated cardiomyopathy at baseline. The cohort reported by Hawwa *et al*.^47^ included 26% with coronary artery disease and 31% with a prior diagnosis of heart failure.

### Left ventricular mass index

Nineteen echocardiographic studies^25^, ^26^, ^27^, ^28^, ^29^, ^30^, ^31^, ^32^, ^33^, ^34^, ^35^, ^36^, ^37^, ^38^, ^39^, ^40^, ^41^, ^42^, ^47^ reported the mean changes in LVMI following transplantation for their entire cohort with 16 reporting significant reductions in LVMI at follow‐up.^25^, ^26^, ^28^, ^29^, ^30^, ^31^, ^33^, ^34^, ^35^, ^36^, ^38^, ^39^, ^40^, ^41^, ^42^, ^47^ The magnitude of change observed varied greatly between studies. Iqbal *et al*.,^34^ in a cohort of 22 participants, reported the largest reduction in LVMI of 131 g/m^2^ three months after transplantation. Three studies found no significant change in LVMI.^27^, ^32^, ^37^

Four echocardiography studies only presented changes in LVMI based on predetermined subgroups.^43^, ^44^, ^45^, ^46^ These two studies examined the effect of baseline LVMI on subsequent changes.^43^, ^46^ In their cohort, Vaidya *et al*.^46^ reported that pre‐transplantation LVMI was the only predictor of subsequent LVMI regression following transplantation [odds ratio 1.50, 95% confidence interval (CI) 1.26–1.80]. Hernandez *et al*.^43^ studied 60 patients, with initial LVMI shown to be an independent predictor of subsequent change in LVMI. Salerno *et al*.^45^ examined changes in LVMI in 104 patients treated with either everolimus or mycophenolate mofetil. While a significant reduction in LVMI from baseline to follow‐up was seen in both groups (everolimus 126.5 ± 46.4 to 121.9 ± 39.4 g/m^2^, *P* < 0.05; mycophenolate 116.6 ± 38.3 to 113 ± 28.9 g/m^2^, *P* < 0.05), there was no significant difference between the groups. Hernandez *et al*.^44^ studied the effect of angiotensin‐converting enzyme polymorphisms. Those with an unfavourable genotype (highest angiotensin‐converting enzyme activity) had a significant increase in LVMI after transplantation (23.3 ± 7.9%; *P* < 0.05), whereas in those with a more favourable genotype, no change was observed (−0.08 ± 4.9%; *P* > 0.05).

All three CMR studies reported no significant overall change in LVMI.^48^, ^49^, ^50^ However, the trends in mean change were conflicting. Patel *et al*.^49^ observed an increase in LVMI in transplant recipients and a decrease in the control group of haemodialysis patients (−3.6 ± 16.7%/year vs. 2.75 ± 9.1%/year). Prasad *et al*.^48^ reported a reduction in LVMI in both recipients and controls who remained on the waiting list (recipients −1.98 ± 5.5 g/m^2^ and controls −0.36 ± 5.7 g/m^2^; *P* = 0.44). The third CMR study by Hayer *et al*.^50^ also reported no significant change in LVMI, from baseline (89 ± 38 to 83 ± 23 g/m^2^; *P* ≥ 0.05).

Two echocardiographic studies and two CMR studies recruited suitable control groups.^27^, ^35^ Keven *et al*.^35^ studied 28 transplant recipients and 23 haemodialysis patients with follow‐up at 1 year. There was a significant reduction of LVMI in transplant recipients from baseline to follow‐up. However, the magnitude of change was not significantly different from that observed in the control group (recipients −10 ± 24 g/m^2^ vs. controls −5.6 ± 22 g/m^2^; *P* > 0.05). De Lima *et al*.^27^ studied 36 haemodialysis patients and 17 transplant recipients at mean follow‐up of 30 ± 8 months. In both groups, no significant change was observed in LVMI from baseline to follow‐up [LVMI (g/m^2^) recipients 156.7 ± 51.3 to 132.9 ± 31.0, *P* > 0.05; controls 170.6 ± 50.8 to 155.6 ± 43.1, *P* > 0.05]. The two CMR studies reported no significant overall change in LVMI following transplantation compared with the control group.^48^, ^49^

**Figure 2:**
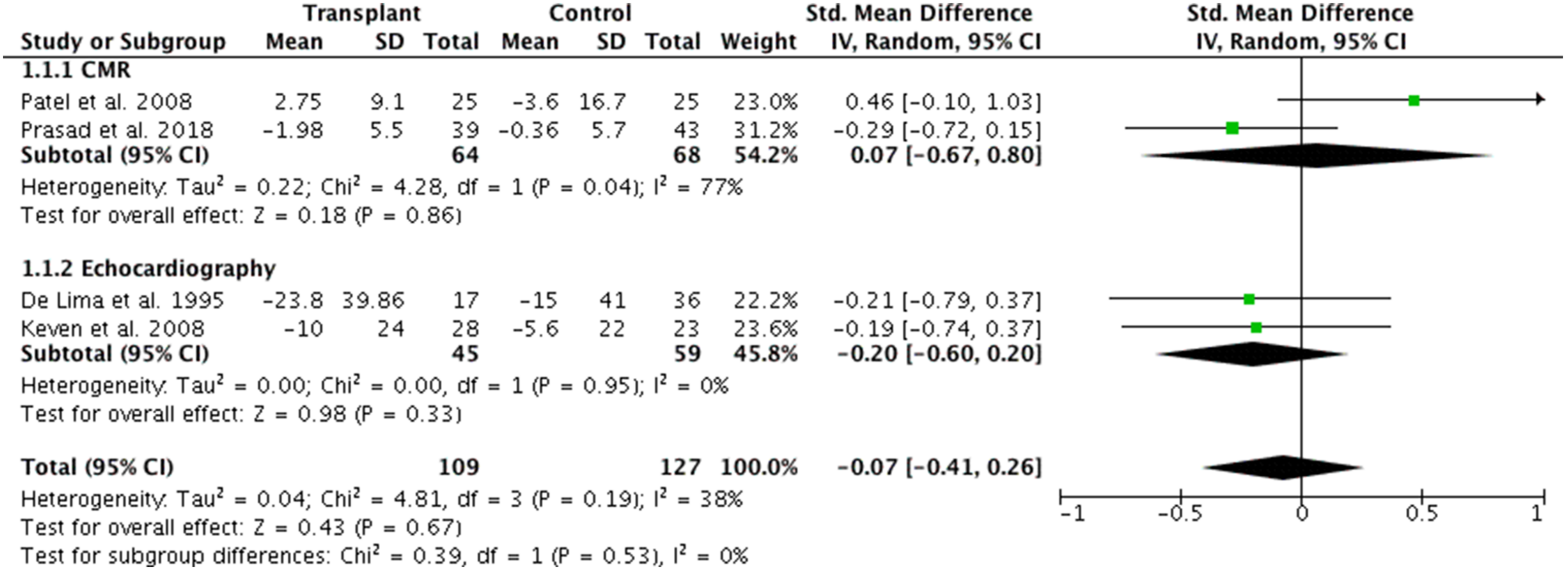
Meta‐analysis of changes in left ventricular mass index following renal transplantation. Subgroup analysis presented based on imaging modality. CI, confidence interval; CMR, cardiac magnetic resonance imaging; IV, inverse variance; SD, standard deviation.

A meta‐analysis was conducted of the four studies reporting change in LVMI in transplant recipient and controls (*Figure* *2*). A total of 236 participants were included in this analysis; the overall SMD was −0.07 (95% CI −0.41 to 0.26), *P* = 0.67, suggesting no difference between transplant and control groups. However, heterogeneity was moderate (*I* ^2^ = 38%). This was regarded as low‐quality evidence using the GRADE approach due to low participant numbers and heterogeneity between the studies included. Subgroup analysis is also presented based on imaging modality. The two echocardiographic studies^27^, ^35^ [SMD −0.20 (95% CI −0.60 to 0.20); *P* = 0.33] and the two CMR studies^48^, ^49^ [SMD 0.07(95% CI −0.67 to 0.80)] showed no mean change in LVMI. There was no significant difference between the findings of the two imaging modalities (*P* = 0.53). However, heterogeneity in the echocardiographic subanalysis was low (*I* ^2^ = 0%) but substantial (*I* ^2^ = 77%) in the CMR subanalysis.

### Systolic function

Fifteen studies reported changes in left ventricular ejection fraction (LVEF) across their whole cohort: 11 using echocardiography^25^, ^28^, ^29^, ^30^, ^31^, ^32^, ^36^, ^39^, ^41^, ^42^, ^47^ and three using CMR.^48^, ^49^, ^50^ None of the studies using echocardiography included a control group. Eight studies^25^, ^28^, ^30^, ^32^, ^36^, ^39^, ^42^, ^47^ reported significant increases in LVEF following transplantation, six of which had recruited individuals with normal mean LVEF.^25^, ^30^, ^32^, ^36^, ^39^, ^42^ Deng *et al*.^28^ recruited 48 participants with a mean LVEF of 40 ± 11%, which increased to 60 ± 14% (*P* < 0.05). Hawwa *et al*.^47^ also reported that in participants with reduced LVEF, significant improvements were observed following transplant (ejection fraction 41 ± 10% to 50 ± 12%; *P* < 0.0001).

**Figure 3:**
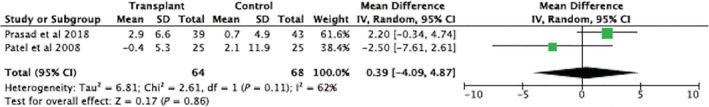
Meta‐analysis of cardiac magnetic resonance imaging studies representing change in left ventricular ejection fraction after renal transplant. CI, confidence interval; IV, inverse variance; SD, standard deviation.

One CMR study by Hayer *et al*.^50^ reported a significant improvement in LVEF from baseline to follow‐up (ejection fraction 68 ± 9% to 73 ± 9%; *P* < 0.05). However, when comparing changes to control participants with ESKD, both Patel *et al*.^49^ and Prasad *et al*.^48^ reported no statistically significant change in LVEF. Meta‐analysis of these two studies, consisting of 64 transplant recipients and 68 control participants receiving regular dialysis, showed no overall change in LVEF in transplant recipients compared with controls [mean difference 0.39% (95% CI −4.09% to 4.87%); *P* = 0.86] with high heterogeneity (*I* ^2^ = 62%) (*Figure* *3*). The quality of evidence (GRADE) was rated as very low quality due inconsistency between the results, the low numbers of trials included, and overall participant numbers.

### Left ventricular dimensions

**Figure 4:**
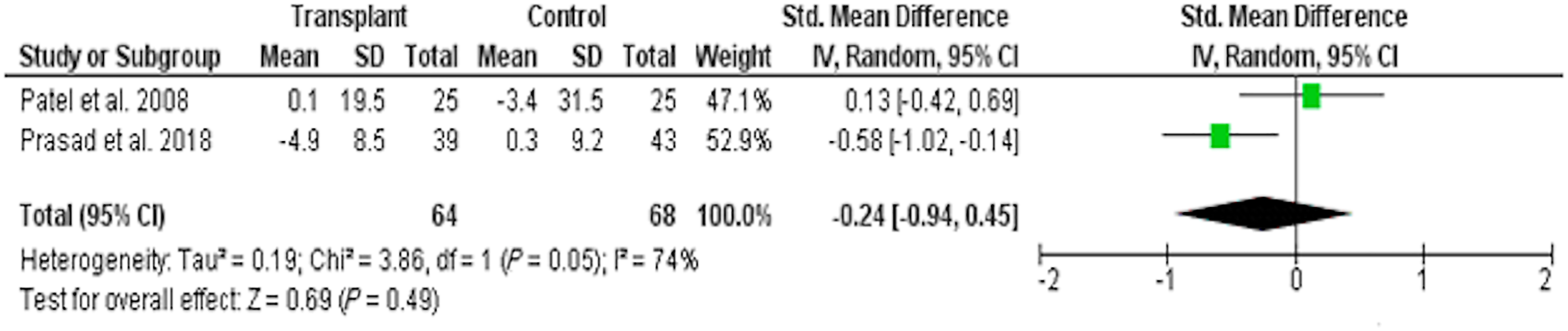
Meta‐analysis of cardiac magnetic resonance imaging studies representing change in end‐diastolic volume after renal transplant. CI, confidence interval; IV, inverse variance; SD, standard deviation.

The most reported measure was left ventricular internal diameter in diastole in 13 non‐controlled echocardiographic studies with all but three reporting a significant reduction.^25^, ^29^, ^31^, ^32^, ^33^, ^34^, ^36^, ^37^, ^40^, ^41^, ^42^, ^43^, ^47^ All three CMR studies reported left ventricular end‐diastolic volume (LVEDV) with Hayer *et al*.^50^ reporting significant reduction from baseline (79 ± 24 to 63 ± 20 mL/m^2^; *P* < 0.05). Conflicting results however were observed in the other two studies with follow‐up of control groups. Prasad *et al*.^48^ reported a reduction in LVEDV compared with controls (recipients  −4.9 ± 8.5 mL/m^2^ vs. controls 0.3 ± 9.2 mL/m^2^; *P* = 0.02), whereas Patel *et al*.^49^ reported no significant difference in mean percentage change (controls −3.4 ± 31.5% vs. recipients 0.1 ± 19.5%; *P* = 0.64). Meta‐analysis of these CMR studies also highlighted that there were high levels of heterogeneity (*I* ^2^ = 74%) and that there was no overall significant change in LVEDV following transplantation [SMD −0.24 (95% CI −0.94 to 0.45); *P* = 0.49] (*Figure* *4*). The quality of evidence (GRADE) was rated as very low quality; this was again due to inconsistency between the results of the two included trials and low participant numbers.

### Diastolic dysfunction

The most reported parameter of diastolic dysfunction was E/A ratio with three studies reporting statistically significant changes following transplantation.^25^, ^27^, ^28^ One controlled study by De Lima *et al*.^27^ reported a small reduction in E/A ratio (1.42 ± 0.6 to 1.10 ± 0.4; *P* < 0.05) at 1 year follow‐up, whereas Deng *et al*.^28^ reported a small increase (1.04 ± 0.57 to 1.21 ± 0.52; *P* = 0.001). An *et al*.^25^ reported that recipients with moderate diastolic dysfunction (Grade 2) before transplantation showed a significant reduction in E/A ratio at 12 months (baseline 1.13 vs. 0.98; *P* < 0.05), whereas those with mild dysfunction (Grade 1) only exhibited a significant change at the 5 year follow‐up (baseline 0.72 vs. 0.81 at 5 years; *P* < 0.05).^34^ Mitral valve deceleration time was also reported in four studies,^28^, ^29^, ^31^, ^42^ with one study reporting a small significant increase^42^ and one a small significant decrease.^28^ Neither of these changes represented a change in the grade of diastolic function observed.

## Discussion

Reversing uraemic cardiomyopathy is potentially the key to reducing cardiovascular morbidity and mortality in ESKD. Although no targeted therapy has been shown to achieve this, it is generally assumed that restoration of kidney function by kidney transplantation reverses the changes observed. At present, however, the evidence does not support this.

We have shown that the majority of uncontrolled echocardiographic studies reported significant reductions in LVMI. However, making conclusions based on these data is problematic. Echocardiography is unreliable when measuring LVMI due to inaccuracy where large volume fluctuations occur.^17^ CMR is more accurate and reproducible and is accepted as the gold‐standard imaging modality for patients with ESKD.^7^ None of the three CMR studies included in our review found a significant change in LVMI. Furthermore, in a meta‐analysis of the four available studies with control groups, renal transplantation was not associated with any reduction in LVMI, and subgroup analysis indicated that this finding was not affected by imaging modality. This analysis also clearly highlights that none of the controlled studies, regardless of imaging modality, reported significant changes in LVMI following transplantation.

A similar pattern was also observed in left ventricular function, with the majority of echocardiographic non‐controlled longitudinal studies reporting significant improvements in LVEF following transplant. This finding is also supported by the work of Wali *et al*.^51^ where 102 transplant recipients with left ventricular dysfunction showed significant improvement at 1 year when assessed with radionuclide ventriculography. However, this study was not included in the systematic review as LVMI was not considered. Among the three CMR studies, the patterns of change observed were conflicting. Hayer *et al*.^50^ report a significant change in recipients from baseline to follow‐up, whereas both Patel *et al*. and Prasad *et al*. did not. In addition, there was also no convincing evidence that successful renal transplantation improves diastolic left ventricular function. It would, therefore, appear that the assumption that the features of uraemic cardiomyopathy are reversed by successful renal transplantation is not supported by the current published literature.

Before concluding that uraemic cardiomyopathy is irreversible, it is important to examine the quality of the evidence available. Studies were generally classified as poor with only two rated as good and two as fair using the Newcastle–Ottawa scoring system. In addition, the assessment of the evidence across studies for each comparison (GRADE) ranged from ‘very low quality’ to ‘low quality’. The majority were opportunistic and unblinded, with little attempt to reduced risk of systematic bias. Only four studies, comprising a total of 109 transplant recipients, recruited a suitable control group, which was followed up.^27^, ^35^, ^48^, ^49^ A further limitation was the lack of sample size justification with no studies powered to detect a change in LVMI. Previous work, however, has indicated that to detect a change in left ventricular mass of 10 g with 90% power using 2D and M‐mode echocardiography, 78 and 162 participants would be required, respectively. As a result, only five echocardiographic studies included in the review can be considered to have sufficient power to reliably detect clinically significant changes in left ventricular mass.^52^ The number required to detect the same change using CMR is much smaller, with only 13 participants required indicating that all three CMR studies recruited adequate numbers of participants.^52^ The fact that only three CMR studies have been conducted, with a total of 88 transplant recipients included, is a major weakness of the current evidence base. The meta‐analyses also demonstrated high heterogeneity, suggesting that the currently available studies do not reliably answer the question of whether uraemic cardiomyopathy is reversible. Some of this may be explained by many studies appearing to have an opportunistic design that is examining patients that happened to have a heart scan performed before and after transplantation with consequential bias, rather than being prospectively designed.

While there are weaknesses in the evidence base, it may also be true that uraemic cardiomyopathy is not reversible. Indeed, the presented meta‐analysis looking at controlled studies, including those using the gold‐standard technique of CMR, suggests that this might well be the case. Following renal transplantation, many traditional risk factors for cardiovascular disease persist and in some cases may develop *de novo*.^53^ Hypertension, dyslipidaemia, and diabetes are all recognized complications of both steroids and calcineurin inhibitors, which are routinely administered following transplant. In addition, there is also persistence of non‐traditional risk factors including uraemia, proteinuria, and chronic inflammation.^53^ Transplantation cannot fully reverse these factors, which may explain the persistence of uraemic cardiomyopathy.

Our study has several strengths in that it included data from both echocardiography and CMR studies, which enabled all relevant data pertaining to the subject to be incorporated. The number of studies identified ensured that there was a significant pooled sample size on which conclusion could be based, although with 26 studies identified, the number of participants was only 1998, highlighting the fact that many studies were very small. There were, however, significant limitations. While there were an appropriate number of studies included in the systematic review, the number suitable for meta‐analysis was small with only four studies eligible. There were also moderate levels of heterogeneity noted among the studies when meta‐analysis was undertaken. Subsequent sensitivity analysis suggested that this was being driven by the conflicting findings of the CMR studies. Such heterogeneity can make the interpretation of any findings difficult. However, we took the view that demonstrating this variability between studies highlights the need for further work to be conducted in this area. Another limitation of the studies included is the short length of follow‐up. It therefore cannot be concluded that uraemic cardiomyopathy might be reversible in the longer term with no study having more than 12 month follow‐up. Furthermore, because we used the assessment of LVMI as the primary selection criteria, studies looking at other important features such as longitudinal strain and right heart changes were not systematically examined.

## Conclusions

Reversing uraemic cardiomyopathy is a potential target for reducing the cardiovascular morbidity and mortality associated with chronic kidney disease. This syndrome has generally been assumed to be reversible by renal transplantation. Our review has highlighted that at present, it is unclear if this is true.

This review also highlights the need for adequately powered and controlled studies to answer this fundamental question and provides further insights into other potential strategies to reverse uraemic cardiomyopathy and improve the increased cardiovascular risk associated with ESKD.

## Conflict of interest

The authors report no relationships that could be construed as a conflict of interest.

## Funding

This work was supported by the British Heart Foundation Clinical Research Training Fellowships (FS/18/29/33554 to L.C.P., FS/16/73/32314 to A.M.P., and FS/19/16/34169 to J.P.L.).

## Author contributions

C.J.F. and L.C.P were responsible for the concept and design of the review. L.C.P. and J.P.L. performed the literature search and data extraction. L.C.P. and A.R. performed the methodological quality analysis using the Newcastle–Ottawa score and the GRADE system. L.C.P. performed the data analysis and was the primary author of the manuscript. All authors were involved in the preparation and editing of the final manuscript.
